# Correspondence

**Published:** 1867-10

**Authors:** 


### Correspondence.

PHILADELPHIA, Pa., August 1st, 1867.

EDITOR OF CHICAGO MEDICAL JOURNAL:—

Proposing to write from time to time, letters containing accounts of the various clinics, etc., of this city, I commence my series with an account of a case recently brought to my notice, and which is of no little interest.

On *July 4th*, I was called to visit Mrs. E. M., aged 26 years. Her husband informed me that they were married July 1st, and on the subsequent night he had endeavored to have connection with her, but found the hymen prevented. After this attempt, she "at once commenced bleeding from the vagina," and this hemorrhage had not ceased when I was called to see her. These are the facts, as gained from the husband.

I found the patient anæmic, extremities cold, with body hot; countenance pallid; eye congested; tongue furred, white; pulse quick, but excessively weak; and complaining of severe pain, "*swelling*" in the belly. Upon questioning her as to her *menstrual life*, I found she had always been regular as to *time* of appearance, but the "show" had continued sometimes two weeks, and never less than one, during which there was a constant, but very *slow oozing of blood*. She observed that she had been "regularly unwell" two weeks before, and that she was "confident that her husband had laçerated her," thus producing this hemorrhage. I at once resorted to an examination, and found she had been freely using preparations of iron, as a local styptic. This, together with clotted blood, was all removed by sol. of sodæ bicarb., and then I found the cause of the trouble. Here was a hymen, consisting of a thick fold of mucous membrane, forming a complete circular septum, only perforated in the centre by a very small round opening. Through this opening, there oozed, slowly, thick, dark blood. This hymen was excessively sensitive, the simple application of a camel's hair pencil giving exquisite pain. It was Impossible to insert my little finger through the opening, first, because of the extreme sensitiveness of the parts, and, secondly, on account of the firm rigidity of the fold. The patient was now completely exhausted, and, having no fear of the hemorrhage, I gave her a full anodyne, and left her until

*July 5th*, when I found her calmed, and her pulse stronger, with the bleeding as yesterday. I now put her under the influence of an anæsthetic, and even *now* I was unable to insert any finger but my little one, and this with only the greatest exertion. On introducing my finger, blood flowed freely. I now divided the hymen in seven distinct points, and then introducing one finger at a time, I succeeded in inserting three, when I broke up the septum thoroughly. The blood, which had until now flowed sluggishly, came forth with a gush, offensive, dark, and grumous. The parts having been washed freely, and the sudden flow of blood subsided, I applied a dressing of equal parts of equal parts of olive oil and lime-water, and gave the patient a grain of opium. At night I found her free from pain, having slept quietly for three hours. The hemorrhage was scanty, but of a better consistency and color. Gave her another opiate.

*July 6th.* Parts healing kindly; bleeding stopped; and sensitiveness very much lessened. Bowels costive; ordered an enema.

*July 7th.* Bowels freely opened. Ordered an injection of cool water for vagina. Greatly improved.

*July 11th.* Parts healed. Introduced my three fingers without giving any pain. Patient walking about, *well.*

This has been an interesting case, for several reasons, not the least of which is, that which shows that here was a patient with a slow and painfully tedious menstruation during her whole menstrual life—a space of twelve years—and during each period suffering very much pain, and having an offensive discharge of blood, all of which had been caused by this impenetrable hymen, and this hemorrhage which commenced after the attempt at coition on July 1st, was but her courses, brought on prematurely by excitement. To-day I saw the patient, and she says, "for twelve years I have never known four weeks so free from pain as those which have just passed." She was taken "unwell the 27th of July, and remained so for three days, having a free discharge of *bright blood*, and have been free from pain." Yours,

E. R. HUTCHINS, M.D.

---

EDITOR OF CHICAGO MEDICAL JOURNAL,

*Dear Sir:*—I notice that your colleague, and our distinguished fellow-citizen and professional brother, Dr. Freer, in the letter which you have given us, *verbatim et literatim*, in the September number of the JOURNAL, alludes, though not in the most flattering terms, to the practice pursued by European surgeons, in their management of joint-diseases, and draws some comparisons, which, though they might be "odious" to them, must certainly be very gratifying to us.

We might deem the Doctor too severe in his criticism, had not others testified to the want of skill manifested by our transatlantic brethren in this regard.

Dr. C. F. Taylor, of New York City, now in Europe, writes that "they are at least twenty years behind the age in the treatment of Potts' Disease, relying entirely upon the various preparations of iron and the recumbent posture" in their treatment of this malady.

And that this statement will apply with equal force to their management of joint-diseases generally, any reader of the *London Lancet* may safely infer, on perusal of Mr. John Hilton's "Lectures on Pain, and the Therapeutic Influence of Mechanical and Physiological Rest in Accidents and Surgical Diseases," published in that journal in 1863. This eminent English surgeon advocates the employment of the recumbent position and absolute rest of the affected joint in all articular diseases, and appears perfectly satisfied with the result which must necessarily follow such a course of treatment—provided the patient survives it all—*viz.:* complete anchylosis.

What well-informed American surgeon, at the present day, relies upon recumbency in the management of these diseases?

Thanks to American ingenuity and skill, a better and more scientific method of overcoming diseased action in these cases has been discovered. With the affected part thoroughly protected by suitable apparatus, the patient is enabled to enjoy the pleasures and benefits of free exercise in the open air, an advantage of incalculable value, and one which, in many cases, cannot be dispensed with.

The principle of extension and counter-extension for removing the pressure from diseased articular surfaces, and obviating the constant tendency to contraction and subsequent structural shortening of the muscles, is now employed, not only in the treatment of disease (inflammation) of the hip-joint, but also in the same condition occurring in other articulations, and with the happiest results, patients almost invariably recovering with the integrity of the affected joint unimpaired.

It is a circumstance of which the American people, and we especially, as members of the medical profession, in America, may justly be proud, that greater advancement has been made in orthopædic surgery in this country within the last ten or fifteen years, than in the old world during the past two centuries. The most useful discoveries and important practical suggestions regarding these matters have been made by American physicians.

It may interest your readers to know that Dr. Freer's suggestion that "Europeans could learn something regarding mechanical surgical treatment" from their "American cousins," is just now being acted upon.

Dr. Taylor, (before alluded to) is now in Europe, for the purpose of securing European recognition of American inventions for the treatment of deformities, and has, thus far, met with very flattering success. Yours, respectfully,

F. O. EARLE.

*84 Washington St.*

